# Study on the Sulfuration Mechanism of Concrete: Microstructure and Product Analysis

**DOI:** 10.3390/ma13153386

**Published:** 2020-07-31

**Authors:** Ditao Niu, Yao Lv, Xiguang Liu, Lei Chen, Guoxin Chen, Binqiang Zhang

**Affiliations:** 1 School of Civil Engineering, Xi’an University of Architecture and Technology, No.13 Yanta Rd., Xi’an 710055, China; niuditao@163.com (D.N.); xgliu@xauat.edu.cn (X.L.); cl18706785510@163.com (L.C.); cgx1072046612@163.com (G.C.); binqiangzhang@126.com (B.Z.); 2 State Key Laboratory of Green Building in Western China, Xi’an University of Architecture and Technology, No.13 Yanta Rd., Xi’an 710055, China

**Keywords:** sulfuration mechanism, microstructure, powdery precipitated substances, gypsum, ettringite

## Abstract

This paper presents an experimental investigation of the sulfuration mechanism of concrete. The microstructure, mineral phase composition, substance content, and pH of the concrete were determined using scanning electron microscopy, X-ray diffraction, comprehensive thermal analysis, and pore-solution pH test. It was observed that light-grey spots appeared on the surface of the specimen, and a large amount of powdery precipitated substances appeared. At the initial stage of sulfuration reaction, the formation of ettringite blocked the concrete pores and densified its cracks and voids. Subsequently, ettringite reacted with H^+^ to form gypsum, and the continuous increase in gypsum in the pores increased the number of cracks and broadened their width. Gypsum was the final product of the sulfuration reaction, and the mass percentage of gypsum in the powdery precipitated substances at different water–cement ratios was more than 50%. When the water–cement ratios was 0.37, 0.47, and 0.57, the highest Ca(OH)_2_ content was found for the lowest water–cement ratio. As the water–cement ratios increased, the amount of powdery precipitated substances decreased and the CaCO_3_ content and pH increased.

## 1. Introduction

Coal is one of the most important energy sources in the world. The global coal consumption in 2017 was 3731 Mtoe, which accounted for 28% of the total energy consumption [1]. In China, the reserves of coal are much higher than those of oil and natural gas [2]. The total coal consumption in China in 2018 was 2.74 billion tons, which accounted for 59% of disposable energy [3]. The consumption of sulfur-containing coal led to a large amount of SO_2_ emission from industrial production [4,5]. From 2000 to 2006, the total SO_2_ emission in China increased by 53% from 21.7 to 33.2 million tons [6]. According to the report of the State of the Environment in China, the total SO_2_ emission was almost 20 million tons as of 2014 [7].

Because of the special production process, the SO_2_ concentration in industrial environment is much higher than that in general atmospheric environment. The SO_2_ concentration in Wuhan Iron and Steel Plant was monitored. The SO_2_ concentration of the crystallization-tank section in the coking ammonium sulfate workshop was 6.65 mg/m^3^ [8], and the maximum SO_2_ concentration in Wuhan No. 2 steelmaking plant was 1.29 mg/m^3^ [9].

SO_2_ diffuses into concrete and reacts with calcium-containing substances, which reduces the alkalinity of pore solution and expands the solid-phase volume, that is, concrete sulfuration [10]. On the one hand, the expansion failure of concrete from outside to inside leads to spalling and damage of concrete cover, as well as deterioration of its physical and mechanical properties. On the other hand, the decrease in the pore solution pH results in the destruction of the steel bar passivation film, which accelerates the structural failure of reinforced concrete structures. 

Developing technologies and strategies for sulfur resistance of concrete is imperative in industrial construction. To achieve this target, understanding the sulfuration mechanism of concrete is necessary. The study on sulfuration mechanism of the concrete is the foundation for the prediction model of corrosion depth in SO_2_ environment. Furthermore, the research is beneficial to put forward the evaluation method of concrete durability in SO_2_ environment.

Only a few investigations have been carried out through theoretical research [11], in situ testing [12], and accelerated experiments in the laboratory [13,14,15,16,17,18,19]. Most studies focus on sulfuration depth, and physical and mechanical properties. The mass and compressive strength of sulfated concrete increased at first, and then decreased with the increase of ages [13,14,15]. The sulfuration rate of concrete is influenced by the material properties and environmental parameters. Yu [13] and Niu [15,16] found that the sulfuration depth of concrete increased with the increase of water–cement ratios. Niu [17] found that fly ash concrete was better than ordinary concrete in resisting SO_2_ corrosion. Tang [18] found that the sulfuration depth increased with temperature and SO_2_ concentration. When the relative humidity was in the range of 40–90%, the sulfuration rate of the concrete was maximum at the relative humidity of 80% [18]. Scholl [19] carried out accelerated corrosion test of mortar under the action of SO_2_ and CO_2_. The results show that the sulfuration depth of the mortar was 0.5 mm at the age of 90 days, and the carbonation depth was 6 mm. 

However, there are few studies on the sulfuration mechanism of concrete at present. The study on sulfated products of concrete is the basis step for studying the sulfuration mechanism. Sulfur oxides can react with all the calcium compounds of hydrated cement, and convert them into sulfur-bearing compounds, which mainly come under consideration includes calcium sulfite, calcium sulfate and calcium sulfoaluminate [12].

The types of sulfur-bearing compounds are different under different conditions and situations. The formation and the stability of ettringite (3CaO·Al_2_O_3_·3CaSO_4_·32H_2_O, AFt) are affected by ambient temperature [20,21,22,23]. The increase of temperature in a proper range can promote the formation of AFt [20], and it cannot be formed in the concrete beyond a certain temperature [21,22]. The formed AFt can be separated under the condition of high temperature [23]. The types of calcium sulfate are affected by ambient humidity. Pavlik [12] tested the flue concrete, the dominant product of concrete sulfuration was gypsum at the higher humidity area, while anhydrite at the less humidity area. The pH of the pore solution affects the formation of calcium sulfoaluminate in concrete. It was reported that the disappearance of AFt and 3CaO·Al_2_O_3_·CaSO_4_·12H_2_O (AFm) at 20 °C were at pH ≤10.7 and ≤11.6, respectively [24]. The pH ranges of AFt that could stably exist at 25, 50, and 85 °C were 10.43–12.52, 10.52–12.41, and 10.87–12.25, respectively [25]. 

The diffusion and reaction of SO_2_ in concrete is a complicated physicochemical process. As the age increases, the internal condition of concrete changes. Therefore, the sulfated products may be transformed. No one has verified this through experiments. According to the above-mentioned discussion, the sulfated products and the sulfuration mechanism of concrete were studied in the present work. The microstructure of concrete was observed using scanning electron microscopy (SEM) to explore the crack-development pattern and sulfated product-type changes at different ages. The mass, X-ray diffraction (XRD), comprehensive thermal analysis (TG-DSC), and pH of the powdery precipitated substances were tested to investigate the damage degree and final sulfated products in the concrete. The effects of water–cement ratio on the mass, substance content, and pH in the powdery precipitated substances were discussed.

## 2. Theoretical Chemical Reaction

SO_2_ diffuses into concrete through micro-pores and micro-cracks, and continuously dissolves in water to form H_2_SO_3_. On the one hand, H_2_SO_3_ ionizes into H^+^ and ${\mathrm{SO}}_{3}^{2-}$. On the other hand, H_2_SO_3_ reacts with O_2_ in the air to form H_2_SO_4_, which ionizes into H^+^ and ${\mathrm{SO}}_{4}^{2-}$ [26].

(1) $${\mathrm{SO}}_{2}+H_{2}O\to H_{2}{\mathrm{SO}}_{3}$$

(2) $$H_{2}{\mathrm{SO}}_{3}\to H^{+}+{\mathrm{HSO}}_{3}^{-}$$

(3) $${\mathrm{HSO}}_{3}^{-}\to H^{+}+{\mathrm{SO}}_{3}^{2-}$$

(4) $${2H}_{2}{\mathrm{SO}}_{3}+O_{2}\to {2H}_{2}{\mathrm{SO}}_{4}$$

(5) $$H_{2}{\mathrm{SO}}_{4}\to 2H^{+}{+\mathrm{SO}}_{4}^{2-}$$

H^+^, ${\mathrm{SO}}_{3}^{2-}$, and ${\mathrm{SO}}_{4}^{2-}$ diffuse into concrete through pore solution and react with hydration products in concrete. First, Ca(OH)_2_ ionizes into Ca^2+^ and OH^−^, which react with H^+^, ${\mathrm{SO}}_{3}^{2-}$, and ${\mathrm{SO}}_{4}^{2-}$ to form CaSO_3_, CaSO_4_, and H_2_O. Subsequently, H^+^, ${\mathrm{SO}}_{3}^{2-}$, and ${\mathrm{SO}}_{4}^{2-}$ react with calcium silicate hydrate (C–S–H), and the products are silica gel, CaSO_3_, CaSO_4_, and H_2_O.

(6) $$H^{+}+{\mathrm{OH}}^{-}\to H_{2}O$$

(7) $$2xH^{+}+x\mathrm{CaO}\cdot y{\mathrm{SiO}}_{2}\cdot zH_{2}O\to x{\mathrm{Ca}}^{2+}+y{\mathrm{SiO}}_{2}\cdot zH_{2}O+xH_{2}O$$

(8) $${\mathrm{Ca}}^{2+}{+\mathrm{SO}}_{3}^{2-}\to {\mathrm{CaSO}}_{3}$$

(9) $${\mathrm{Ca}}^{2+}{+\mathrm{SO}}_{4}^{2-}\to {\mathrm{CaSO}}_{4}$$

CaSO_3_ is an intermediate product, and it can combine with O_2_ to form CaSO_4_. CaSO_4_ can combine with water to form hemihydrate gypsum and gypsum.

(10) $${2\mathrm{CaSO}}_{3}+O_{2}\to {2\mathrm{CaSO}}_{4}$$

(11) $${\mathrm{CaSO}}_{3}+{2H}_{2}O\to {\mathrm{CaSO}}_{4}\cdot {2H}_{2}O$$

Gypsum reacts with calcium aluminate in concrete to form calcium sulfoaluminate. When the gypsum content is low, gypsum and calcium aluminate first form AFm.

(12) $$\begin{array}{l} 3\mathrm{CaO}\cdot {\mathrm{Al}}_{2}O_{3}+{\mathrm{CaSO}}_{4}\cdot 2H_{2}O+10H_{2}O\\ \to 3\mathrm{CaO}\cdot {\mathrm{Al}}_{2}O_{3}\cdot {\mathrm{CaSO}}_{4}\cdot {12H}_{2}O \end{array}$$

With the continuous formation of gypsum, AFm is transformed into AFt.

(13) $$\begin{array}{l} 3\mathrm{CaO}\cdot {\mathrm{Al}}_{2}O_{3}\cdot {\mathrm{CaSO}}_{4}\cdot {12H}_{2}O+2\left({\mathrm{CaSO}}_{4}\cdot {2H}_{2}O\right){+16H}_{2}O\\ \to 3\mathrm{CaO}\cdot {\mathrm{Al}}_{2}O_{3}\cdot {3\mathrm{CaSO}}_{4}\cdot {32H}_{2}O \end{array}$$

When the temperature exceeds a certain temperature, AFt decomposes into AFm, and calcium sulfate. When the temperature dropped to a room temperature, AFm could transform to AFt again in moist environment [23].

(14) $$\begin{array}{l} 3\mathrm{CaO}\cdot {\mathrm{Al}}_{2}O_{3}\cdot {3\mathrm{CaSO}}_{4}\cdot {32H}_{2}O\\ \to 3\mathrm{CaO}\cdot {\mathrm{Al}}_{2}O_{3}\cdot {\mathrm{CaSO}}_{4}\cdot {12H}_{2}O+2\left({\mathrm{CaSO}}_{4}\cdot 2H_{2}O\right)+16H_{2}O \end{array}$$

Because of the decrease of pH, AFm and AFt decompose, and the products are gypsum and an aluminum-containing gel [27].

(15) $$\begin{array}{l} 3\mathrm{CaO}\cdot {\mathrm{Al}}_{2}O_{3}\cdot {\mathrm{CaSO}}_{4}\cdot {12H}_{2}O+12H^{+}\\ \to {3\mathrm{Ca}}^{2+}+2{\mathrm{Al}}^{3+}{+\mathrm{CaSO}}_{4}\cdot 2H_{2}{O+16H}_{2}O \end{array}$$

(16) $$\begin{array}{l} 3\mathrm{CaO}\cdot {\mathrm{Al}}_{2}O_{3}\cdot {3\mathrm{CaSO}}_{4}\cdot {32H}_{2}O+12H^{+}\\ \to {3\mathrm{Ca}}^{2+}+2{\mathrm{Al}}^{3+}+3\left({\mathrm{CaSO}}_{4}\cdot 2H_{2}O\right){+32H}_{2}O \end{array}$$

## 3. Materials and Experiments

### 3.1. Materials

P.O. 42.5 Ordinary Portland cement, in compliance with Chinese Standard GB 175-2007 [28], was used in this experiment. The mass percent of cement chemical composition determined by X-ray fluorescence spectroscopy (EDX8000, Shimadzu, Kyoto, Japan) is listed in Table 1. The fine aggregate used in this study had a fineness modulus of 2.34. The coarse aggregate used granite macadam with continuous grading from 5 to 25 mm. A polycarboxylic–based superplasticizer was used in the experiment with water reducing rate of 35%, solid content of 40%, and alkali content of 6.5%. The amount of water reducer was 0.5% of the cementitious material.

### 3.2. Specimen Preparation

The experimental specimens with dimension of 100 mm × 100 mm × 100 mm were prepared using three mixture proportions. The specimens were prepared in three mixtures, namely C40, C30, and C20, with three water–cement ratios of 0.37, 0.47, and 0.57, respectively. The specimens were moisture-cured at 20 ± 2 °C and 95% relative humidity for 28 days and then dry-cured until 90 days. The mixture proportion of concrete are listed in Table 2. According to Chinese Standard CB/T 50081-2019 [29], three specimens of each water–cement ratio were used for compressive strength at the age of 28 days. The compressive strength and its standard deviation are shown in Table 2.

### 3.3. Testing Method

#### 3.3.1. Sulfuration Test

SO_2_ is mainly generated by combustion, and it is emitted after desulfurization treatment through the chimney. The wet-desulfurization technology without Gas Gas Heater (GGH) is usually adopted in China. The SO_2_ concentration and temperature of the flue gas decreased, and the humidity increased. The average temperature of the flue gas was approximately 50 °C, and the humidity at the tail device reached saturation. Therefore, the temperature in this experiment was 50 °C, and the relative humidity was 98%.

**Figure 1:**
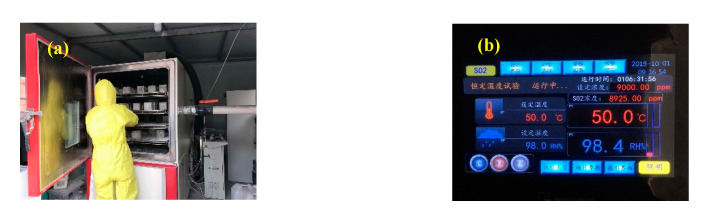
Sulfuration test setup: (**a**) concrete sulfuration test chamber; and (**b**) parameter control panel.

There is no generic standard of concrete sulfuration test method. According to the concrete accelerated carbonation test method in accordance with Chinese Standard GB/T 50082-2009 [30], the accelerated sulfuration test method was established. The self-designed concrete sulfuration test chamber was used in this test, as shown in Figure 1. The test procedure was performed as follows:

- The specimens were taken out and dried at 60 °C for 48 h. When the specimen temperature dropped to room temperature, the specimen surface was sealed with epoxy resin except for the two opposite sides. The purpose is to ensure that the transmission and reaction of SO_2_ in the concrete is one-dimensional.
- The specimens were placed on a bracket in the concrete sulfuration test chamber. The distance between adjacent specimens was not less than 50 mm.
- The ambient temperature in the sulfuration test chamber was 50 °C, the relative humidity was 98%, and the SO_2_ concentration was 9000 ppm.
- The experiments were performed for four different test ages, including 2, 5, 10, and 20 days, and the specimens were taken out from the sulfuration test chamber. The powdery precipitated substances on the specimens surface were collected for 20 days.

#### 3.3.2. SEM

SEM (Quanta 200, FEI, Hillsboro, OR, USA) test was employed to observe the morphology of concrete after the sulfuration reaction. Single specimen was used at one test age for each water–cement ratio. The specimen was cut into slices of 1–2 mm thickness from the surface, and then the samples with the diameter about 5 mm were obtained by pliers and tweezers. A few samples were taken out at different test ages and were immersed in ethanol to terminate all the reactions, including the hydration reaction and carbonation reaction. Before the test, the samples were removed from ethanol, air dried at room temperature for 10 min, and dried at 50 °C for 24 h. A platinum coating was subsequently applied onto the surface of the specimens for enhanced electrical conductivity. The measurements were carried out with the secondary electron detector. The accelerating voltage was 20 kV and the working distance was 10 mm.

#### 3.3.3. Pore-Solution pH Test 

Three specimens were used for each water–cement ratio. The powdery precipitated substances were gathered from the exposed surface of specimens at the age of 20 days. The powdery precipitated substances of three specimens were crushed and mixed evenly.

Pore-solution pH test was performed to determine the pH of the powdery precipitated substances, and it was conducted three times for each water–cement ratio. The experimental method was performed as follows:

- The powdery precipitated substances of sulfated concrete were crushed and mixed, and the powder was then passed through a 0.16-mm sieve.
- The powder was placed in a weighing bottle and dried at 50 °C for 24 h. Then, the weighing bottle was sealed and transferred in a drying and cooled down to room temperature.
- Three grams of dried powder were placed in a triangular flask. A pipette was used to accurately measure 60 mL of distilled water. Next, the distilled water was poured into the triangular flask. The triangular flask was shaken in an oscillator (Xinbode, Tianjin, China) for 10 min. After standing for 24 h, the solution in the triangular flask was filtered using a fast quantitative filter paper.
- The filtrate was tested using a pH meter (ST310, Ohaus, Pine Brook, NJ, USA).

#### 3.3.4. XRD

XRD (D/Max 2200, Rigaku, Tokyo, Japan) analysis was used to examine the mineral-phase composition of the surface powdery precipitated substances. The powdery precipitated substances were first pulverized using an agate mortar, and then passed through an 80-μm sieve. The powder was dried at 50 Rigaku for 24 h. The diffraction used a Cu-Kα source. Scanning was performed from 5° to 45° at a step of 0.02°.

#### 3.3.5. TG-DSC

The TG-DSC (TGA/DSC 2, Mettler Toledo, Zurich, Switzerland) method was used for qualitative and quantitative investigation of the surface powdery precipitated substances. The preparation method of the powder was the same as that of the powder used in the XRD method. The samples were heated in air atmosphere from 30 to 900 °C at a heating rate of 10 °C/min.

## 4. Results and Discussion

### 4.1. Appearance Analysis

**Figure 2:**
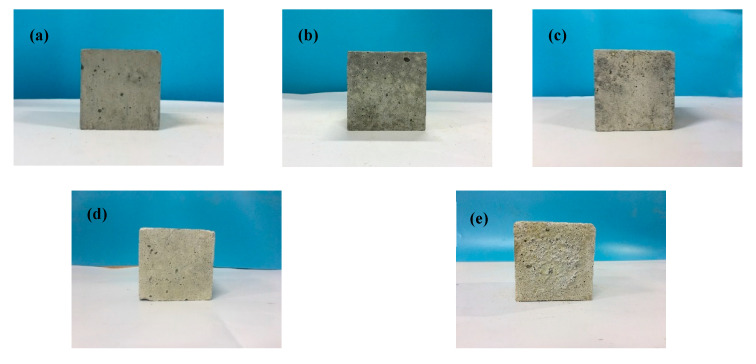
Appearance of C30 specimen at different sulfuration ages: (**a**) 0 days; (**b**) 2 days; (**c**) 5 days; (**d**) 10 days; and (**e**) 20 days.

The appearance of the C30 specimens for sulfuration ages of 0, 2, 5, 10, and 20 days is shown in Figure 2. The changes in the concrete appearance mainly underwent four stages. Firstly, the surface of the hardened concrete was dark grey and smooth, and some capillary pores were visible to the naked eyes (Figure 2a). Then, light-grey spots began to appear on the concrete surface at the initial stage of sulfuration reaction (Figure 2b). The light-grey area on the surface increased with the progress in the sulfuration reaction, and the surface became smoother and denser (Figure 2c). Next, all the areas on the specimen surface became light-grey, and the corners began to peel off (Figure 2d). Finally, a large amount of white powdery precipitated substances appeared on the specimen surface, and the surface became rough (Figure 2e).

The appearance of light-grey spots on the concrete surface was due to the formation of sulfated products. These products gradually increased as the sulfuration progressed, and, therefore, the light-grey area on the concrete surface increased. Because the sulfated products blocked the pores in the concrete, the surface initially became denser. When the sulfated products exceeded the pore capacity, cracks formed on the concrete surface. Subsequently, powdery precipitated substances appeared.

**Figure 3:**
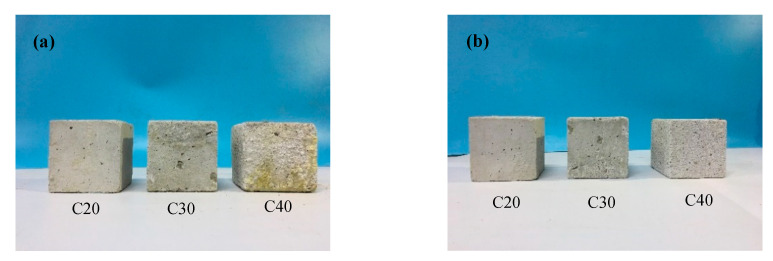
Appearance of sulfated concrete at the age of 20 days with different water–cement ratios: (**a**) before cleaning; and (**b**) after cleaning.

The appearance of concrete at water–cement ratios of 0.37, 0.47, and 0.57 in the 20 days sulfuration is shown in Figure 3a. Only a thin layer of powdery substances appeared on the surface of the C20 specimen, and powdery precipitated substances were produced in part of the C30 specimen. The C40 specimen produced a large amount of powdery precipitated substances throughout. The corrosion layer of the C40 specimen was the thickest, and the expansion volume was the largest.

After cleaning the powdery precipitated substances, the appearance of sulfated concrete for 20 days is shown in Figure 3b. Compared with that shown in Figure 3a, the appearance of the C20 specimen remained almost unchanged. However, the visible pores in the surface of C30 and C40 specimens were larger than that of C20 specimen, and the surface was rough and honeycomb after sulfuration reaction. The change of specimens’ appearance increased with the decrease of water–cement ratios.

The cement content per unit volume of concrete increased with the decrease in the water–cement ratio, thus the content of the hydration products Ca(OH)_2_ increased. Increasing the alkali content can accelerate the reaction rate of concrete [31]. Therefore, the change in the appearance of concrete with the low water–cement ratio was more serious.

### 4.2. Microscopic Fracture Surface Observation

**Figure 4:**
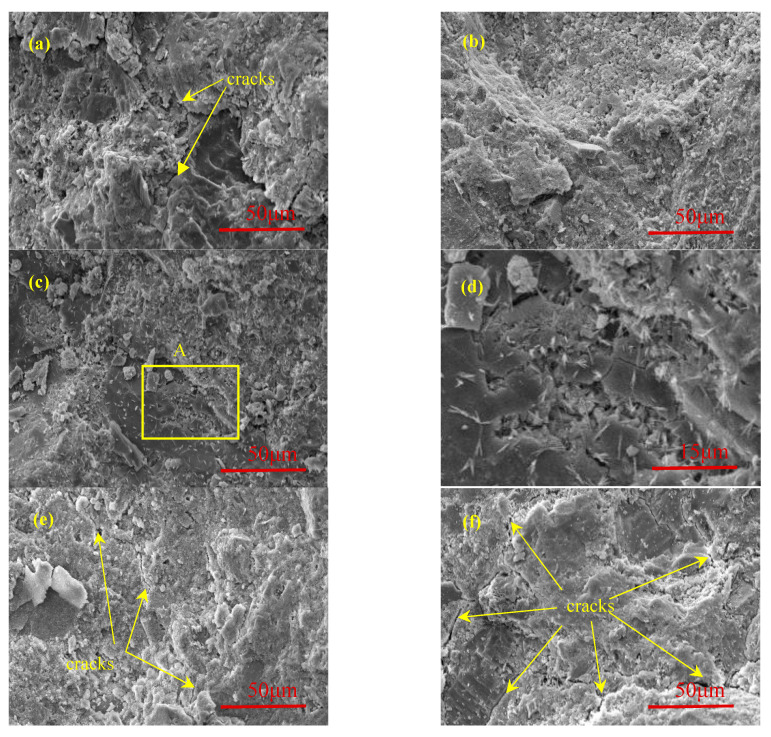
Cracks development of C30 specimen at different sulfuration ages: (**a**) 0 days; (**b**) 2 days; (**c**) 5 days; (**d**) A in (**c**); (**e**) 10 days; and (**f**) 20 days.

The crack development trend of the C30 specimen at different sulfuration ages is shown in Figure 4. An initial damage existed in concrete due to the few cracks and voids (Figure 4a). A large amount of white flocculent substances remained, and the initial cracks were obviously reduced after sulfuration for 2 days (Figure 4b). After sulfuration for 5 days, many white substances were generated inside the concrete (Figure 4c). Figure 4d shows the enlargement of the rectangular area shown in the Figure 4c, and a large amount of needle-like materials can be seen. When the sulfuration ages increased to 10 days, the needle-like products disappeared, and new micro-cracks appeared (Figure 4e). After sulfuration for 20 days, cracks appeared in many locations, and the crack width increased (Figure 4f). 

After sulfuration for 10 and 20 days, a circle of white substances appeared around the cracks, indicating that the cracks were caused by the formation of a large amount of solid sulfated products, which exceeded the capacity of the pore structures. Sulfated products were generated inside the concrete; thus, the solid-phase volume in the cracks and voids continuously increased. The cracks and voids were significantly reduced. Finally, a large amount of sulfated products blocked the pores, resulting in excessive internal stress [16]. Therefore, the concrete cracked, and the products precipitated on the concrete surface.

**Figure 5:**
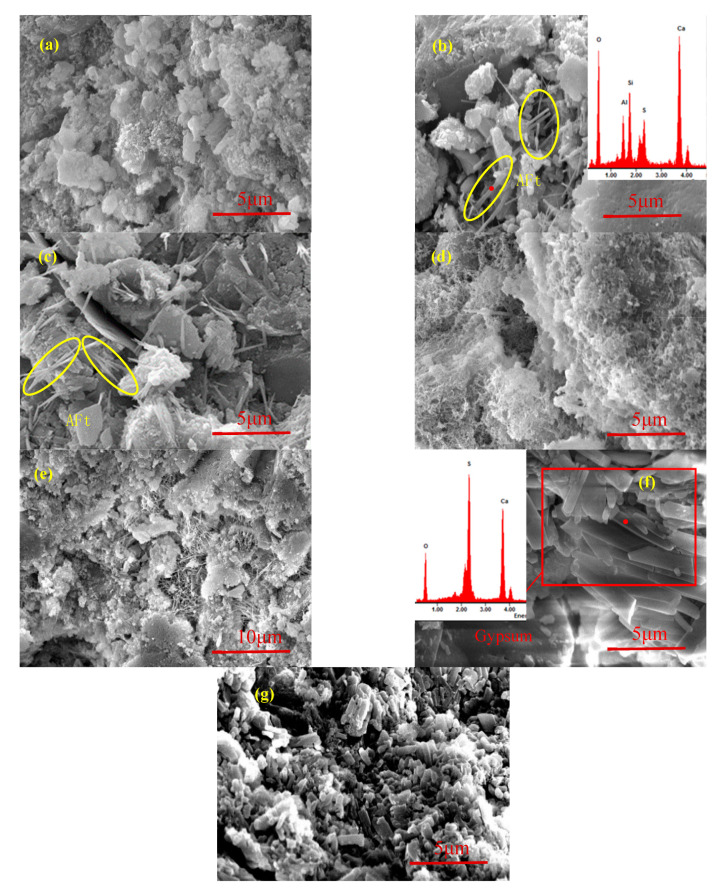
Mineral-phase composition of C30 specimen at different sulfuration ages: (**a**) flocculent hydration products for zero days; (**b**) the formation of AFt for two days; (**c**) the increase of AFt for five days; (**d**) non-gelling silica gels for five days; (**e**) the decomposition of the AFt for 10 days; (**f**) the formation of gypsum for 10 days; and (**g**) a large amount of gypsum for 20 days.

The mineral-phase composition of the C30 specimen at different ages is shown in Figure 5. Many flocculent hydration products appeared in the hardened concrete (Figure 5a). With the diffusion and dissolution of SO_2_ into the concrete, H^+^, ${\mathrm{SO}}_{4}^{2-}$, and ${\mathrm{SO}}_{3}^{2-}$ reacted with the hydration products at the cracks and pores to form a needle-like substance (Figure 5b). The main constituent elements of the needle-like substance were Ca, S, Al, O, and Si; therefore, the needle-like substance was AFt. With the progress in the sulfuration reaction, an increasing amount of needle-like products appeared near the hydration products (Figure 5c), and AFt was continuously formed. 

C–S–H reacted with H^+^ dissolved in water to form non-gelling silica gels, which were stacked with one another (Figure 5d). The cementitious materials in the specimen ran off, and Ca(OH)_2_ was gradually consumed. Then, the pH of the pore solution rapidly decreased, resulting in the decomposition of the AFt crystal. Subsequently, the number of large pores increased (Figure 5e). At the same time, AFt was decomposed by H^+^ to form a plate-like substance (Figure 5f). The main elements were S, Ca, and O, indicating that this type plate crystal was gypsum. Finally, gypsum was produced in large quantity (Figure 5g).

### 4.3. Powdery Precipitated Substances Analysis

To qualitatively and quantitatively study the degree of damage with different water–cement ratios, we determined the mass, composition, and pH of powdery precipitated substances.

#### 4.3.1. Mass

**Figure 6:**
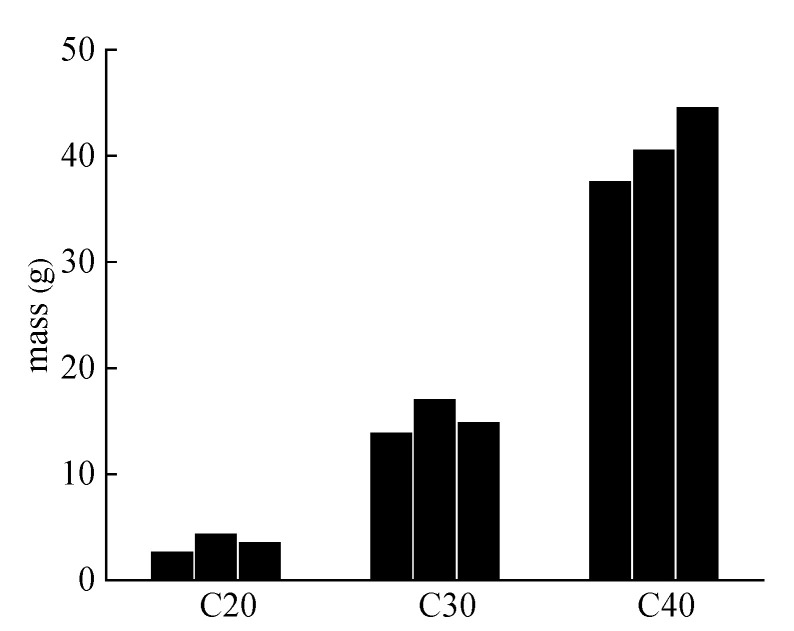
The mass of the powdery precipitated substances at different water–cement ratios. Three samples were tested for each water–cement ratio.

Figure 6 shows the mass of the powdery precipitated substances at different water–cement ratios. The average mass of powdery precipitated substances in the C20, C30 and C40 specimens was 3.67, 15.40, and 41.03 g, respectively. Concrete with a high water–cement ratio had larger and more pores, which played the role of a capacity to absorb expansion caused by the sulfated products [32]. Therefore, the specimen with lower water–cement ratio was more likely to crack. Cracks can further accelerate the diffusion and reaction of SO_2_. Therefore, the mass of powdery precipitated substances increased with the decrease of water–cement ratios.

#### 4.3.2. Composition

**Figure 7:**
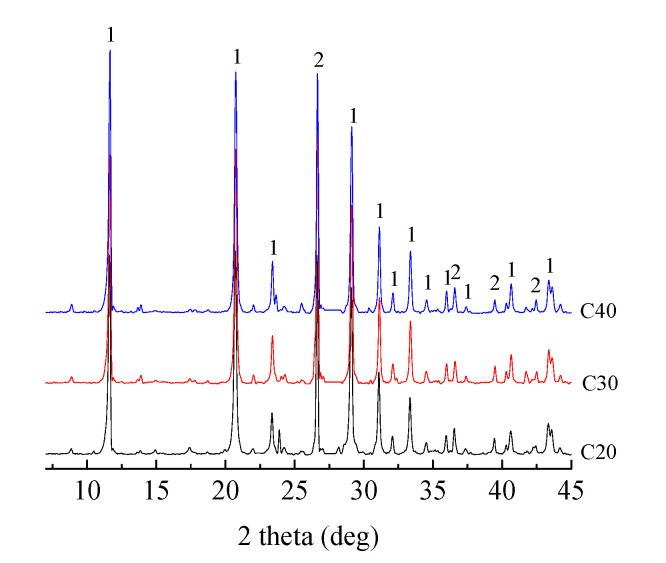
XRD patterns of the powdery precipitated substances at different water–cement ratios: 1, gypsum (CaSO_4_ 2H_2_O); and 2, quartz (SiO_2_).

The composition of the powdery precipitated substances was analyzed by XRD, as shown in Figure 7. Two types of obvious peaks exist in the XRD pattern as is seen through the comparative analysis of the corresponding characteristic angles. The characteristic angles of gypsum are 11.62°, 20.72°, 23.36°, 29.10°, 31.10°, 33.34°, 35.96°, 40.62°, and 43.34°. The characteristic angles of quartz (SiO_2_) are 26.62°, 36.54°, and 39.44°. Therefore, the sulfated product of the powdery precipitated substances was mainly gypsum.

**Figure 8:**
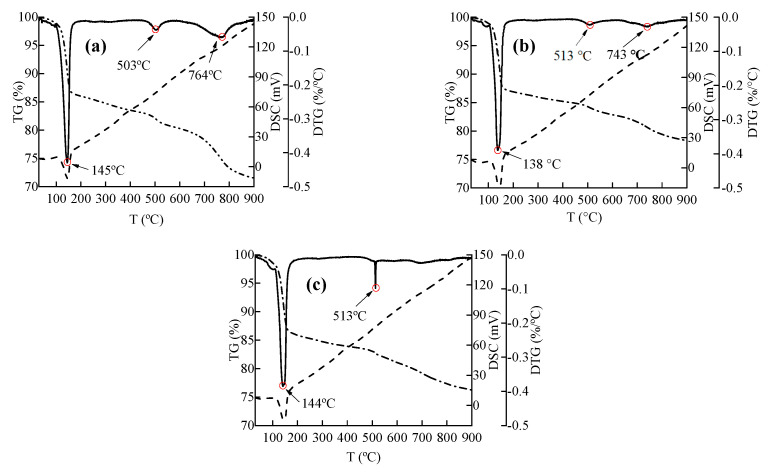
Thermal-analysis curves of the powdery precipitated substances at different water–cement ratios: (**a**) C20 specimen; (**b**) C30 specimen; and (**c**) C40 specimen.

Figure 8 shows the thermal analysis curves of the powdery precipitated substances at three water–cement ratios. Three obvious endothermic peaks were observed before 900 °C, and they represent the dehydration decomposition of three substances. The temperatures of the endothermic peaks were mainly 130–150, 500–520, and 740–770 °C. Related studies have shown that the three temperature ranges correspond to gypsum, Ca(OH)_2_, and CaCO_3_, respectively.

The endothermic peak of gypsum was the most obvious, indicating that the content of gypsum in the powdery precipitated substances was the highest. Gypsum dehydrates and decomposes into hemihydrate gypsum (CaSO_4_·0.5H_2_O) at 150 °C, and hemihydrate gypsum becomes anhydrous gypsum (CaSO_4_) at 170 °C. The content of gypsum was obtained by chemical reaction, i.e., Equation (17).

(17) $${\mathrm{CaSO}}_{4}\cdot {2H}_{2}O\left(s\right)\to {\mathrm{CaSO}}_{4}\left(s\right)+{2H}_{2}O\left(g\right)$$

Figure 8 shows that the gypsum of the powdery precipitated substances at water–cement ratios of 0.57, 0.47, and 0.37 was decomposed at 101.37–170.76, 106.90–173.76, and 111.23–174.76 °C, respectively. The mass-loss rates of concrete were 12.41%, 11.51% and 11.83%, respectively. The mass percentage of gypsum was calculated using Equation (18), and the gypsum contents in the C20, C30 and C40 specimens were 59.29%, 55.01%, and 56.53%, respectively.

(18) $$W_{2}=W_{1}\frac{M_{{\mathrm{CaSO}}_{4}\cdot {2H}_{2}O}}{2M_{H_{2}O}}$$

where $M_{{\mathrm{CaSO}}_{4}\cdot {2H}_{2}O}$ and $M_{H_{2}O}$ are the relative molecular weights of gypsum and H_2_O, respectively. *W*_1_ and *W*_2_ are the weight percentages of H_2_O and gypsum, respectively.

Based on the reported results, the highest gypsum content was found for the highest water–cement ratio. The content of cement per unit concrete decreased with the increase of water–cement ratios. Therefore, the content of reaction product gypsum was likely to decrease. However, the powdery precipitated substances were mainly composed of gypsum and fine aggregate, and the fine aggregate of the powdery precipitated substances in the C20 specimen was the lowest because of the minimal surface damage. Therefore, the relative gypsum content in the C20 specimen was the highest.

The endothermic peak of Ca(OH)_2_ in the C40 specimens was most obvious, indicating that the content of Ca(OH)_2_ was the highest. Ca(OH)_2_ dehydrates and decomposes into CaO, as shown in Equation (19). The contents of Ca(OH)_2_ in the C20, C30 and C40 specimens were 9.85%, 8.81%, and 11.42%, respectively.

(19) $$\mathrm{Ca}{\left(\mathrm{OH}\right)}_{2}\left(s\right)\to \mathrm{CaO}\left(s\right)+H_{2}O\left(g\right)$$

According to the research results, the highest Ca(OH)_2_ content was found for the lowest water–cement ratio. The cement content per unit volume decreased with the increase of the water–cement ratios. Therefore, the content of Ca(OH)_2_ per unit concrete decreased. However, fine aggregate of the C20 specimen was less than that of C30 specimen. Therefore, the Ca(OH)_2_ content of C20 specimen was slightly larger.

CaCO_3_ was observed in the C20 and C30 specimens, and the endothermic peak of CaCO_3_ could not be obviously detected in the C40 specimens. CaCO_3_ decomposes into CaO and CO_2_, as shown in Equation (20). The content of CaCO_3_ in the C20 and C30 specimens was 16.68% and 8.25%, respectively. The carbonation of concrete occurred in air, and the larger was the water–cement ratio, the easier was the carbonation reaction. Therefore, the content of CaCO_3_ increased with the increase of water–cement ratios.

(20) $${\mathrm{CaCO}}_{3}\left(s\right)\to \mathrm{CaO}\left(s\right)+{\mathrm{CO}}_{2}\left(g\right)$$

The main components of the powdery precipitated substances were gypsum, SiO_2_, Ca(OH)_2_, and CaCO_3_. SiO_2_ is the main component of sand and granite, and Ca(OH)_2_ is the hydration product of concrete. CaCO_3_ is the carbonation product formed by the reaction of Ca(OH)_2_ with CO_2_. Therefore, the final sulfated product was gypsum.

#### 4.3.3. Pore-Solution pH

**Figure 9:**
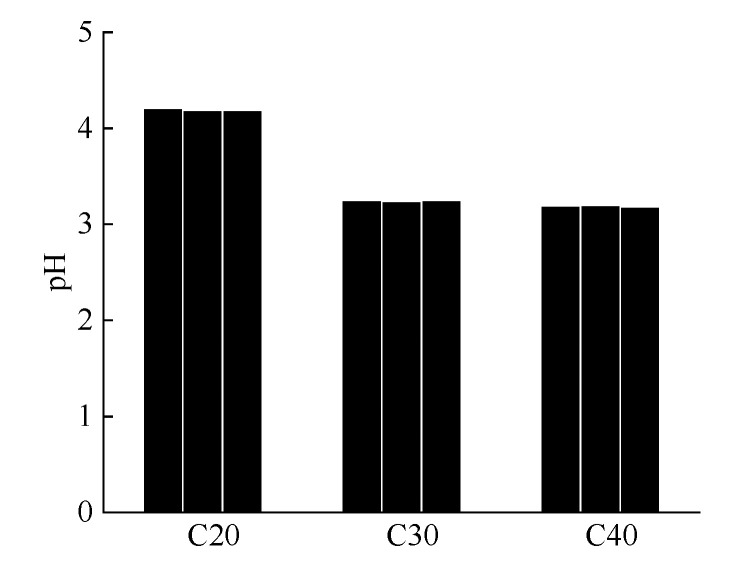
The pH of the powdery precipitated substances at different water–cement ratios. Three samples were tested for each water–cement ratio.

Figure 9 shows the pH of the powdery precipitated substances at different water–cement ratios. The average pH of the powdery precipitated substances in C20, C30, and C40 specimens was 4.20, 3.25, and 3.19, respectively. The specimen appearance at a lower water–cement ratio was rougher and more honeycomb, thus it had a larger specific surface area and could absorb more H^+^ [33]. Therefore, the pH of concrete with a lower water–cement ratio was likely to be lower. However, it can be seen in Section 4.3.2 that the alkaline substances Ca(OH)_2_ were the most in C40 specimens. Therefore, the pH of C20 specimen was much higher than that of C30 and C40 specimens. The pH of C40 specimen was just a little lower than that of C30 specimen.

The pH of the powdery precipitated substances at different water–cement ratios was acidic; thus, it was difficult for calcium sulfoaluminate to exist. Therefore, obtaining AFt and AFm in the powdery precipitated substances was impossible.

## 5. Conclusions

In this study, the variations in the appearance, development trend of a micro-crack, product-type changes, and powdery precipitated substances of sulfated concrete were evaluated. The main conclusions are as follows:

(1) The surface of hardened concrete was dark grey and smooth, and light-grey spots appeared on the surface at the initial stage of sulfuration reaction. Then, the light-grey area increased, and the surface increasingly became denser. Next, all areas on the specimen surface became light grey, and the corners of the concrete began to fall off. Finally, a large amount of powdery precipitated substances appeared. The content of the powdery precipitated substances decreased with the increase in the water–cement ratio.

(2) The initial cracks in the hardened concrete were obviously reduced because of the formation of sulfated products. The number of cracks initially decreased with the sulfuration age. Following the sulfuration-reaction process, the products exceeded the capacity of the pores in the concrete; thus, new cracks appeared. In the later stage of sulfuration reaction, the continuous increase in solid products in the pores increased the number of cracks and broadened their width.

(3) AFt was formed at the initial stage of sulfuration reaction. The hydration products in the concrete decomposed by H^+^. C–S–H was decomposed into a non-gelling silica gel. Ca(OH)_2_ constantly dissolved and reacted; thus, the alkalinity of the concrete decreased. Finally, AFt decomposed into gypsum.

(4) The average mass of powdery precipitated substances in the C20, C30, and C40 specimens was 3.67, 15.40, and 41.03 g, respectively. The pH increased with the increase in the water–cement ratio, and the pH were between 3.19 and 4.20. The final product of the powdery precipitated substances was gypsum, and the mass percentages of gypsum at different water–cement ratios were more than 50%. The highest Ca(OH)_2_ content was found for the lowest water–cement ratio, and the CaCO_3_ content increased with the increase in the water–cement ratios.

## Figures and Tables

**Table 1:** Chemical composition of cement (/wt%).

Chemical Composition	CaO	SiO_2_	Al_2_O_3_	Fe_2_O_3_	MgO	Na_2_O	K_2_O	SO_3_	MnO	TiO_2_	SrO	Cr_2_O_3_	ZnO
Content	56.97	23.60	6.05	2.67	2.88	0.38	1.08	5.26	0.60	0.30	0.10	0.08	0.03

**Table 2:** Mixture proportion and compressive strength of concrete.

Specimen	W/C	C/kg∙m^−3^	FA/kg∙m^−3^	CA/kg∙m^−3^	W/kg∙m^−3^	R/kg∙m^−3^	28 Days CS/MPa
C40	0.37	468	704	1055	173	2.34	40.37 (1.41%)
C30	0.47	368	744	1115	173	1.84	32.06 (2.17%)
C20	0.57	304	769	1154	173	1.52	24.40 (0.74%)

W/C, water–cement ratio; C, cement; FA, fine aggregate; CA, coarse aggregate; W, water; R, water reducing; CS, compressive strength.
